# Influence of the mesenchymal stromal cell source on the hematopoietic supportive capacity of umbilical cord blood-derived CD34^+^-enriched cells

**DOI:** 10.1186/s13287-021-02474-8

**Published:** 2021-07-13

**Authors:** Sara Bucar, André Dargen de Matos Branco, Márcia F. Mata, João Coutinho Milhano, Íris Caramalho, Joaquim M. S. Cabral, Ana Fernandes-Platzgummer, Cláudia L. da Silva

**Affiliations:** 1 Department of Bioengineering and iBB – Institute for Bioengineering and Biosciences, Instituto Superior Técnico, Universidade de Lisboa, Lisboa, Portugal; 2 Associate Laboratory i4HB - Institute for Health and Bioeconomy, Instituto Superior Técnico, Universidade de Lisboa, Lisboa, Portugal; 3 Hospital São Francisco Xavier, Centro Hospitalar de Lisboa Ocidental, Lisboa, Portugal; 4 Instituto Gulbenkian de Ciência, Oeiras, Portugal

**Keywords:** Umbilical cord blood, Human hematopoietic stem/progenitor cells, Mesenchymal stromal cells, Bone marrow, Adipose tissue, Umbilical cord matrix, Ex vivo expansion

## Abstract

**Background:**

Umbilical cord blood (UCB) is a clinically relevant alternative source of hematopoietic stem/progenitor cells (HSPC). To overcome the low cell number per UCB unit, ex vivo expansion of UCB HSPC in co-culture with mesenchymal stromal cells (MSC) has been established. Bone marrow (BM)-derived MSC have been the standard choice, but the use of MSC from alternative sources, less invasive and discardable, could ease clinical translation of an expanded CD34^+^ cell product. Here, we compare the capacity of BM-, umbilical cord matrix (UCM)-, and adipose tissue (AT)-derived MSC, expanded with/without xenogeneic components, to expand/maintain UCB CD34^+^-enriched cells ex vivo.

**Methods:**

UCB CD34^+^-enriched cells were isolated from cryopreserved mononuclear cells and cultured for 7 days over an established feeder layer (FL) of BM-, UCM-, or AT-derived MSC, previously expanded using fetal bovine serum (FBS) or fibrinogen-depleted human platelet lysate (HPL) supplemented medium. UCB cells were cultured in serum-free medium supplemented with SCF/TPO/FLT3-L/bFGF. Fold increase in total nucleated cells (TNC) as well as immunophenotype and clonogenic potential (cobblestone area-forming cells and colony-forming unit assays) of the expanded hematopoietic cells were assessed.

**Results:**

MSC from all sources effectively supported UCB HSPC expansion/maintenance ex vivo, with expansion factors (in TNC) superior to 50x, 70x, and 80x in UCM-, BM-, and AT-derived MSC co-cultures, respectively. Specifically, AT-derived MSC co-culture resulted in expanded cells with similar phenotypic profile compared to BM-derived MSC, but resulting in higher total cell numbers. Importantly, a subpopulation of more primitive cells (CD34^+^CD90^+^) was maintained in all co-cultures. In addition, the presence of a MSC FL was essential to maintain and expand a subpopulation of progenitor T cells (CD34^+^CD7^+^). The use of HPL to expand MSC prior to co-culture establishment did not influence the expansion potential of UCB cells.

**Conclusions:**

AT represents a promising alternative to BM as a source of MSC for co-culture protocols to expand/maintain HSPC ex vivo. On the other hand, UCM-derived MSC demonstrated inferior hematopoietic supportive capacity compared to MSC from adult tissues. Despite HPL being considered an alternative to FBS for clinical-scale manufacturing of MSC, further studies are needed to determine its impact on the hematopoietic supportive capacity of these cells.

## Background

Umbilical cord blood (UCB) has emerged as an alternative source of hematopoietic stem/progenitor cells (HSPC) for patients lacking a suitable donor in the context of allogeneic hematopoietic cell transplantation (HCT). Over the last two decades, the number of allogeneic HCT more than duplicated in the United States of America (USA). Considering unrelated donor allogeneic HCT only, the number of UCB transplants in adults peaked at 14% in 2010 but has been losing ground to the adult tissue sources of HSPC (mobilized peripheral blood and bone marrow (BM)) and accounted for 7% of the more than 5000 unrelated donor grafts performed in the USA in 2019 [1]. It is estimated that around 800,000 and 4 million UCB units are currently stored in public or private UCB banks, respectively, worldwide. This investment and development in UCB banking allowed a reduction in the searching time for unrelated donors, as compared to adult tissue sources [2]. Despite being a readily available source, with lower immunogenicity and lower risk of development of graft-vs-host disease (GVHD) compared to other sources, the low cell dose in a single UCB unit constitutes a major limitation [3]. In this context, the majority of HCT with UCB cells were initially limited to children weighing 20–40 kg [4]. To overcome the limitations of a low cell dose for transplantation of adult patients, two main strategies have been employed: (i) HCT using two unmanipulated UCB units (standard of care) [5] and (ii) HCT using two UCB units, one of which containing cells that were expanded ex vivo [6]. Several protocols were developed to promote ex vivo expansion of UCB HSPC, including the use of different media, cytokines, growth factors, and more recently the use of small molecules and chemical compounds [6–8]. Alongside these approaches, BM mesenchymal stromal cells (MSC) have been used in a co-culture system to support the ex vivo expansion and maintenance of HSPC. This strategy emerged to recapitulate the hematopoietic niche within the BM, where MSC have a pivotal role by giving structural support for HSPC to grow but also to influence their homing, stemness and differentiation potential [8]. Over the last years, we have studied the supportive capacity of BM MSC to UCB HSPC ex vivo in a co-culture setting [9–11] with a tailored cytokine cocktail recently established [12]. In a clinical setting, a significant improvement in neutrophil and platelet engraftment was observed in patients with hematologic cancers who received a unit of UCB previously expanded with BM MSC in addition to an unmanipulated UCB unit [13]. Co-transplantation of both BM MSC and UCB HSPC has also been employed in an HCT context with pediatric patients. Infusion of MSC proved to be safe, was associated with decreased incidence of acute GVHD, and consequently reduced transplant-related mortality [14, 15].

From a hematopoietic niche perspective, the use of BM MSC can be seen as the logical choice to establish a recreation of the microenvironment where HSPC reside in vivo. Despite the majority of studies employing human MSC use BM-derived cells [16], requiring an invasive procedure that entails risks to donors, MSC can also be efficiently isolated from other tissues [17]. Overall, adipose tissue (AT) and umbilical cord matrix (UCM) display advantages over BM as a source of MSC, namely ease of collection using minimally/non-invasive procedures. For instance, AT MSC, derived from the stromal vascular fraction (SVF) of AT, can be easily obtained through enzyme-based isolation procedures from subcutaneous AT [18], which is usually discarded as medical waste and offers the possibility of resampling. Interestingly, stromal cells in the SVF share similarities with those of the BM [19] and some studies have focused on the potential of these cells to support ex vivo expansion of UCB progenitors [20–22]. On the other hand, the umbilical cord tissue, specifically the Wharton’s jelly or matrix (UCM), has been explored as a promising source of MSC [23]. Of notice, Wharton’s jelly MSC has been recently proposed as a preferable feeder layer (FL) choice for UCB HSPC expansion ex vivo considering the microenvironment of the umbilical cord and placenta, where UCB hematopoietic progenitors reside in, which differs from the adult BM niche [24]. Although these alternative sources of MSC have been compared with the standard BM-derived MSC, namely focusing on identity criteria such as immunophenotype and multilineage differentiation potential [25, 26], a direct comparison among these tissue sources is still missing in what concerns their capacity to support the ex vivo expansion of UCB HSPC.

Regardless of the MSC source chosen for the co-culture system, the main goal would be the development of a cost-effective, clinical-grade, co-culture system using serum-free (SF)/xenogeneic-free (XF) culture materials towards the maximization of cell yield, while increasing product consistency and maintaining product features [12]. Specifically, the translation of such system to an approved cell therapy product would certainly rely on two main parameters that are commonly evaluated in clinical trials: total nucleated cells (TNC) and percentage of CD34^+^ cells. This is due to the fact that higher doses of TNC, as well as UCB units enriched with CD34^+^ cells, have been positively correlated with better clinical outcomes (namely, neutrophil engraftment) in patients receiving expanded UCB cells [6]. For single UCB transplantation, a minimum TNC dose of ≥ 2.5 × 10^7^/kg and a minimum prefreeze CD34^+^ dose of ≥ 1.5 × 10^5^/kg is desirable [27].

To fulfill the existing gap regarding the efficacy of using alternative sources to BM-derived MSC, we designed this study whose aim is to make a comprehensive comparison of the ex vivo expansion capacity of UCB CD34^+^-enriched cells in a co-culture system using different sources of MSC, namely BM, AT, and UCM. In an attempt to establish a XF co-culture system, we also tested the feasibility of establishing FL of MSC from the different sources using medium supplemented with fibrinogen-depleted human platelet lysate (HPL), instead of fetal bovine serum (FBS). FBS is the most widely used serum-based supplement for the culture of eukaryotic cells in vitro, being rich in a variety of components, such as vitamins, hormones, transport proteins, and growth factors, that allows cell growth, maintenance, and proliferation in vitro. Although FBS raises issues related to safety (e.g., high endotoxin content, potential source of microbial contaminants and presence of xenogeneic serum antigens that can trigger severe immunological reactions) and animal welfare [28], it is still the most popular supplement used for MSC manufacturing, including at a clinical level [13]. Specifically, a phase III clinical trial with patients that received HSPC expanded ex vivo in a co-culture system with MSC previously cultured using FBS was recently completed (ClinicalTrials.gov Identifier: NCT01854567).

## Methods

### Human samples

Human samples were obtained from local hospitals (umbilical cord blood (UCB) and tissue: Hospital São Francisco Xavier, Centro Hospitalar de Lisboa Ocidental; bone marrow (BM): Instituto Português de Oncologia Francisco Gentil, Lisboa; Adipose tissue (AT): Clínica de Todos-os-Santos, Lisboa) under collaboration agreements with Institute for Bioengineering and Biosciences, Instituto Superior Técnico (iBB-IST), after written and informed consent and according to the Directive 2004/23/EC of the European Parliament and of the Council of 31 March 2004 regarding standards of quality and safety for the donation, procurement, testing, processing, preservation, storage, and distribution of human tissues and cells (Portuguese Law 22/2007, June 29), with the approval of the Ethics Committee of the respective clinical institution. All samples used in this study were obtained from human donors (the mothers, in the case of UCB and tissue) that have previously tested negative for common virus and diseases.

### Preparation of human mesenchymal stromal cell (MSC)-feeder layers (FL)

Cells from a single donor of each tissue source (BM, AT and UCM) were used to establish FL, mimicking an allogeneic universal donor for each source, as recently proposed by our group [12]. Human MSC were obtained from the Stem Cell Engineering Research Group (SCERG) cell bank, at iBB-IST, Lisboa, Portugal. These cells were previously isolated and expanded under normoxia conditions using fetal bovine serum (FBS)-supplemented medium, characterized and cryopreserved by our group according to established protocols [29]. Cells from all tissue sources used in this study have been previously shown to comply with International Society for Cell & Gene Therapy (ISCT) criteria in what concerns identity and characterization of MSC (i.e., expression of CD73, CD90 and CD105; lack of expression of CD34, CD45, CD73, CD80, CD90, CD105, CD14 and HLA-DR and confirmation of tri-lineage differentiation potential) [30, 31]. Firstly, MSC were thawed and seeded using low glucose Dulbecco’s Modified Eagle’s Medium (DMEM) (Thermo Fisher Scientific, United States of America (USA)) supplemented with 10% (v/v) MSC-qualified FBS (Thermo Fisher Scientific) (i.e., specially tested to support the expansion and clonal enumeration (MSC CFU-F assay) of MSC). Then, in order to establish MSC-based FL under xenogeneic-free (XF) conditions, i.e., MSC expanded without FBS-containing medium, half of the cells were subjected to two adaptive passages with low glucose DMEM supplemented with 5% (v/v) fibrinogen-depleted human platelet lysate (HPL) (UltraGRO^TM^-PURE; kindly provided by AventaCell Biomedical Corp., USA) (Certificate of Analysis (CoA) provided), while the other half continued to be expanded in low glucose DMEM with 10% MSC-qualified FBS (both supplemented with 1% (v/v) Antibiotic-Antimycotic (A/A) (Gibco, USA)). MSC were seeded at 3000 cells/cm^2^ into cell culture flasks and medium was changed every 3 days until 80–90% confluence was reached. After the two adaptive passages, MSC were seeded (in P5 or P6) onto wells of a 12-well plate using the appropriate medium. Once confluence was reached, MSC growth was arrested by using medium supplemented with 0.5 μg/mL (BM and AT MSC) [32] or 5 μg/mL (UCM MSC) ([33]; unpublished results) Mitomycin-C (Sigma-Aldrich, USA) for 2.5–3 h at 37 °C and 5% CO_2_. A higher concentration of Mitomycin-C was used for UCM MSC due to their higher in vitro proliferative capacity compared to their adult counterparts [23]. Mitomycin-C treated FL were carefully washed twice and kept with the respective medium at 37 °C and 5% CO_2_ for no more than 72 h until further co-culture with hematopoietic stem/progenitor cells (HSPC).

### Isolation of umbilical cord blood (UCB) mononuclear cells (MNC)

MNC were isolated from fresh UCB samples through a Ficoll (GE Healthcare, USA) density gradient centrifugation. After washing with 2 mM ethylenediamine tetraacetic acid (EDTA) (Sigma-Aldrich) in phosphate-buffered saline (PBS) (Sigma-Aldrich) and upon treatment with ammonium chloride (155 mM) (Sigma-Aldrich) for 10 min at 4 °C to eliminate residual erythrocytes, MNC were cryopreserved using Recovery Cell Culture Freezing Medium (Gibco) and stored in a liquid/vapor phase nitrogen tank.

### Enrichment for CD34^+^ cells

Cryopreserved MNC from three UCB samples were individually thawed in DMEM + 20% (v/v) FBS and washed with magnetic-activated cell sorting (MACS) buffer. CD34^+^ HSPC were then isolated using the CD34 MicroBead Kit UltraPure (Miltenyi Biotec, Germany) through MACS, according to the manufacturer’s instructions. In order to attain a highly pure CD34^+^ cell population, cells from the positive fraction were subjected to a second LS MACS column.

### Ex vivo expansion of CD34^+^-enriched cells

**Fig. 1:**
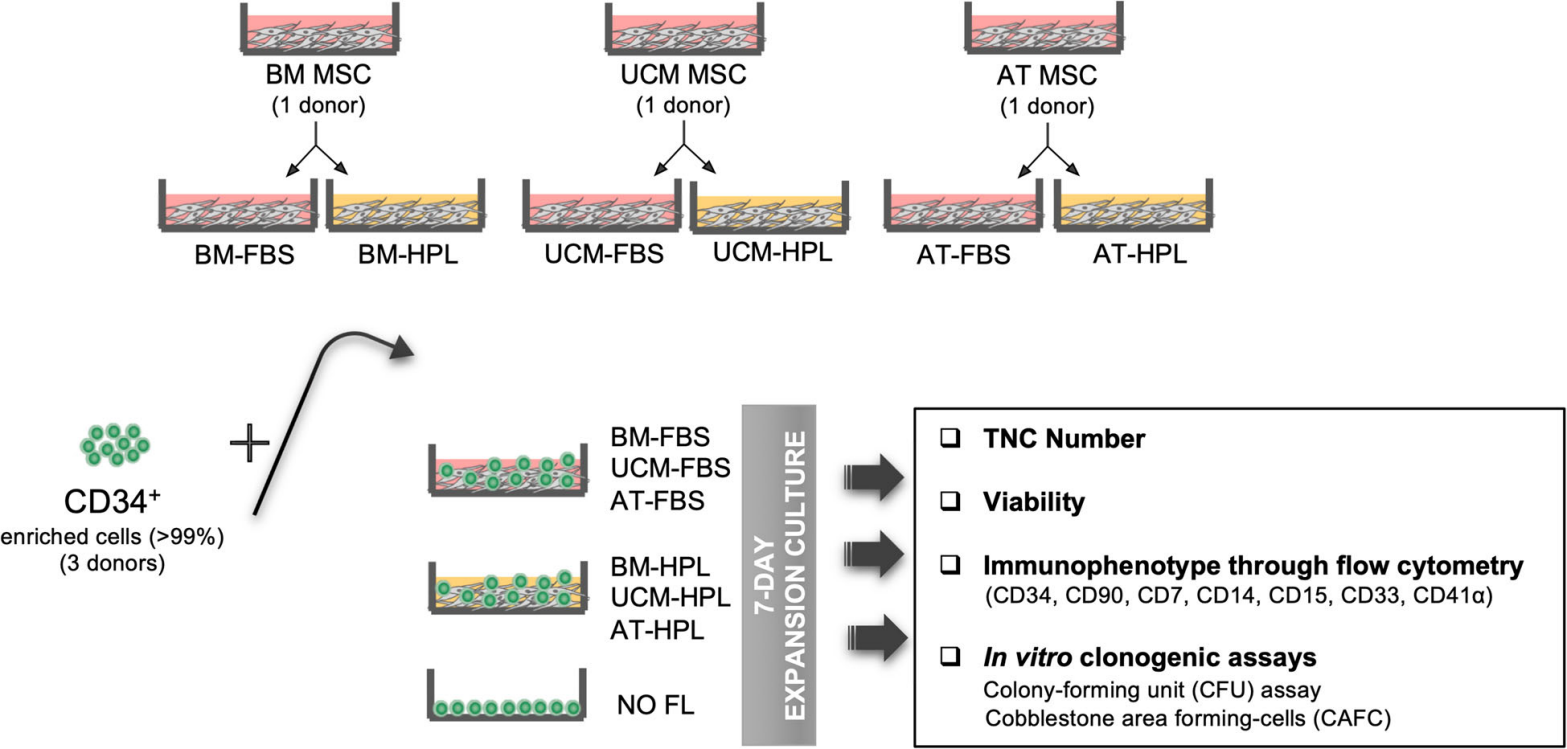
Schematic representation of the experimental design. Umbilical cord blood CD34^+^ cells were expanded in a co-culture system using a mesenchymal stromal cell (MSC) feeder layer (FL) from different sources for 7 days. MSC were previously expanded using medium with either FBS or HPL. UCB cells were characterized by immunophenotyping and in vitro clonogenic assays. AT, adipose tissue; BM, bone marrow; FBS, fetal bovine serum; FL, feeder layer; HPL, human platelet lysate; TNC, total nucleated cells; UCM, umbilical cord matrix

CD34^+^-enriched cells from UCB (30,000/mL) were resuspended in StemSpan SFEM II medium (STEMCELL Technologies, Canada) supplemented with 1% (v/v) A/A and SCF, TPO, FLT3-L, and bFGF cytokines (PeproTech, USA) (90, 77, 82 and 5 ng/mL, respectively, for co-cultures with a MSC FL; and 64, 80, 61, and 5 ng/mL, respectively, for cultures without a MSC FL). Cytokine concentrations were previously optimized for these culture systems by our group [12]. Two milliliters of cell suspension was deposited in each well of a 12-well plate containing a MSC FL previously prepared as mentioned above (or under stroma-free conditions, i.e., no feeder layer (NO FL)) and expanded for 7 days at 37 °C and 5% CO_2_ in a humidified atmosphere (Fig. 1). At the end of the experiment, UCB total nucleated cell (TNC) count and viability were determined through the Trypan Blue (Gibco) exclusion method.

### Immunophenotypic characterization

HSPC before and after expansion (days 0 and 7, respectively) were firstly incubated with Far Red LIVE/DEAD Fixable Dead Cell Stain Kit (Thermo Fisher Scientific) to assess cell viability and then surface stained with the following anti-human antibodies: CD34 (8G12) PerCP-Cy5.5, CD41α (HIP8) PE (BD Pharmingen, USA); CD90 (5E10) PE, CD7 (CD7-6B7) FITC, CD14 (M5E2) FITC, CD15 (HI98) PE, and CD33 (WM53) PE (BioLegend, USA). Cells were acquired on a FACSCalibur flow cytometer (BD Biosciences, USA), and data was analyzed using FlowJo v10 software (FlowJo LLC, USA).

### In vitro clonogenic assays

The ability of expanded and non-expanded hematopoietic progenitors to proliferate and differentiate was assessed through the colony-forming unit (CFU) assay. Briefly, 1000 (day 0) or 2500 (day 7) cells were resuspended in MethoCult H4434 Classic (STEMCELL Technologies) and seeded onto wells of a 24-well plate. After 14 days of incubation at 37 °C and 5% CO_2_, formed colonies were counted using a bright-field microscope (Olympus CK40-F200, Japan) and classified as erythroid burst-forming unit (BFU-E), colony-forming unit granulocyte-monocyte (CFU-GM) or multilineage colony-forming unit (CFU-Mix). Colony number was normalized by the number of seeded cells and multiplied by the TNC number. Fold-increase (FI) of CFU was calculated dividing the number of colonies on day 7 by the number of colonies on day 0.

Stemness of expanded and non-expanded cells was also assessed through the cobblestone area-forming cells (CAFC) assay. Two thousand cells were resuspended in MyeloCult™ medium (STEMCELL Technologies) supplemented with 350 ng/mL of hydrocortisone (STEMCELL Technologies), seeded on top of a growth-arrested FL of MS-5 cells on a 24 well-plate, in duplicates, and incubated for 14 days at 37 °C and 5% CO_2_. CAFC were visualized using a phase-contrast microscope (Leica DMI3000 B, Germany) and registered if at least 5 cells with cobblestone-like morphology were able to migrate beneath the murine FL [34]. FI of CAFC was calculated dividing the number of CAFC on day 7 by the number of CAFC on day 0.

### Statistical analysis

Statistical analysis was performed using SPSS Statistics v26 software (IBM, USA). Results are presented as mean ± standard error of the mean (SEM). The Shapiro-Wilk test was carried out to assess normal distribution. Paired sample t tests were conducted to compare differences between conditions. A p value < 0.05 was considered statistically significant for all tests.

## Results

### Adipose tissue (AT) mesenchymal stromal cells (MSC) outperform umbilical cord matrix (UCM) MSC in promoting the ex vivo expansion of umbilical cord blood (UCB)-derived hematopoietic stem/progenitor cells (HSPC)

**Fig. 2:**
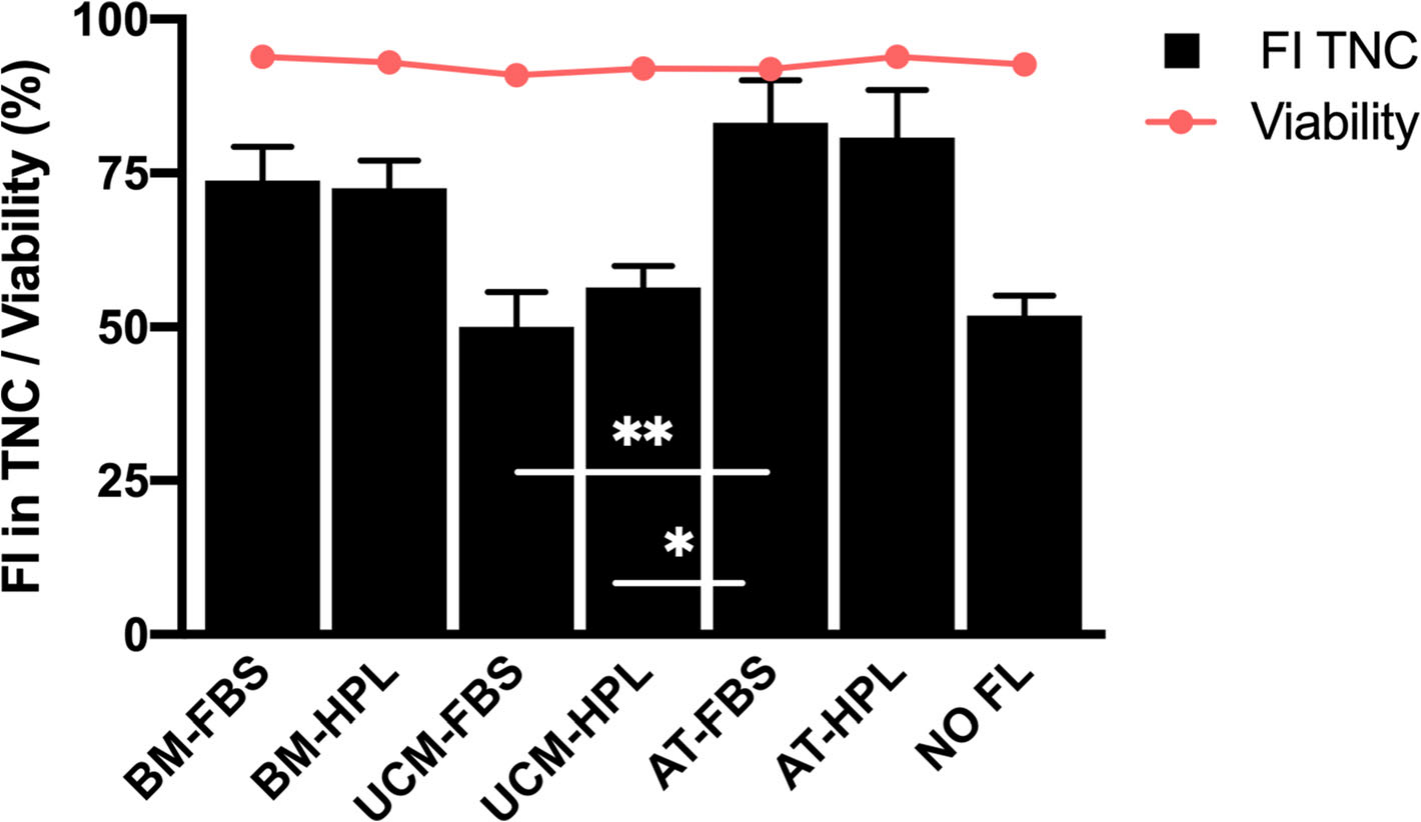
Viability and fold increase in total nucleated cells of expanded umbilical cord blood-derived hematopoietic stem and progenitor cells after 7 days of co-culture with mesenchymal stromal cells from different sources. AT, adipose tissue; BM, bone marrow; FBS, fetal bovine serum; FI, fold increase; FL, feeder layer; HPL, human platelet lysate; TNC, total nucleated cells; UCM, umbilical cord matrix. Values are presented as mean ± SEM. *n* = 3 for all conditions, with exception of ‘NO FL’: *n* = 2. **p < 0.01; *p < 0.05

At day 7 of culture, UCB-derived HSPC showed a high viability (> 90%) in all co-cultures tested using different MSC feeder layers (FL) (bone marrow (BM), AT and UCM), as well as in cultures without a MSC FL (NO FL). Fold increase (FI) in total nucleated cells (TNC) ranged from 50 to 83 (Fig. 2). Co-culture with AT and BM MSC resulted in the highest FI of TNC for UCB cells, with the former allowing slightly higher values (> 80 and > 70, respectively). In the conditions of our study, UCM-derived MSC resulted in the lowest expansion of UCB cells, with FI TNC values similar to the negative control, i.e., HSPC expanded without a MSC FL. In particular, the expansion levels in TNC for UCB cells co-cultured with a FL of UCM MSC previously established using either fetal bovine serum (FBS)- (UCM-FBS) or fibrinogen-depleted human platelet lysate (HPL)-supplemented medium (UCM-HPL) significantly differed (*p* < 0.01 and *p* < 0.05, respectively) from the FI values obtained with HSPC co-cultured with a FL of AT MSC previously established with FBS-supplemented medium (AT-FBS). Nevertheless, the culture medium in which MSC were previously expanded (FBS vs HPL supplementation) did not seem to have a major impact on the overall expansion of HSPC, as observed by the FI TNC values obtained.

### Tissue source of MSC and xenogeneic-free culture conditions for the establishment of the FL influence the differentiative potential of UCB expanded cells

**Fig. 3:**
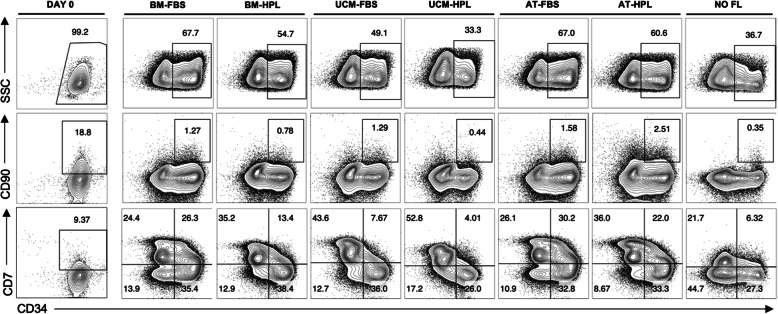
Representative immunophenotypic profile of hematopoietic stem/progenitor cells (HSPC) before and after a 7-day expansion in a co-culture system with mesenchymal stromal cells (MSC) from three different sources (bone marrow, umbilical cord matrix and adipose tissue). Populations are gated on live cells. AT, adipose tissue; BM, bone marrow; FBS, fetal bovine serum; FL, feeder layer; HPL, human platelet lysate; SSC, side scatter; UCM, umbilical cord matrix

**Fig. 4:**
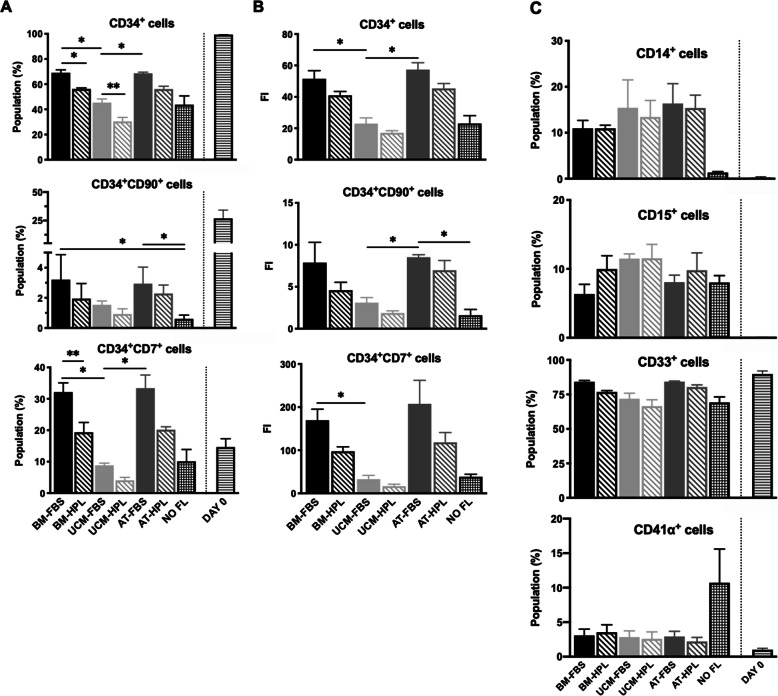
Quantitative characterization of the hematopoietic stem/progenitor cell (HSPC) populations after a 7-day expansion in a co-culture system with mesenchymal stromal cells from different sources. **a** Percentage of hematopoietic stem/progenitor (CD34^+^ cells), more primitive (CD34^+^CD90^+^) and progenitor T cells (CD34^+^CD7^+^) before and after expansion. **b** Fold increase of hematopoietic stem/progenitor (CD34^+^ cells), more primitive (CD34^+^CD90^+^) and progenitor T cells (CD34^+^CD7^+^) after expansion. **c** Percentage of cell populations with myeloid potential before and after expansion. AT, adipose tissue; BM, bone marrow; FBS, fetal bovine serum; FI, fold increase; FL, feeder layer; HPL, human platelet lysate; UCM, umbilical cord matrix. *n* = 3 for all conditions, with exception of ‘NO FL’: *n* = 2. Values are presented as mean ± SEM. **p < 0.01; *p < 0.05 (statistical significance is only showed for FBS conditions)

**Fig. 5:**
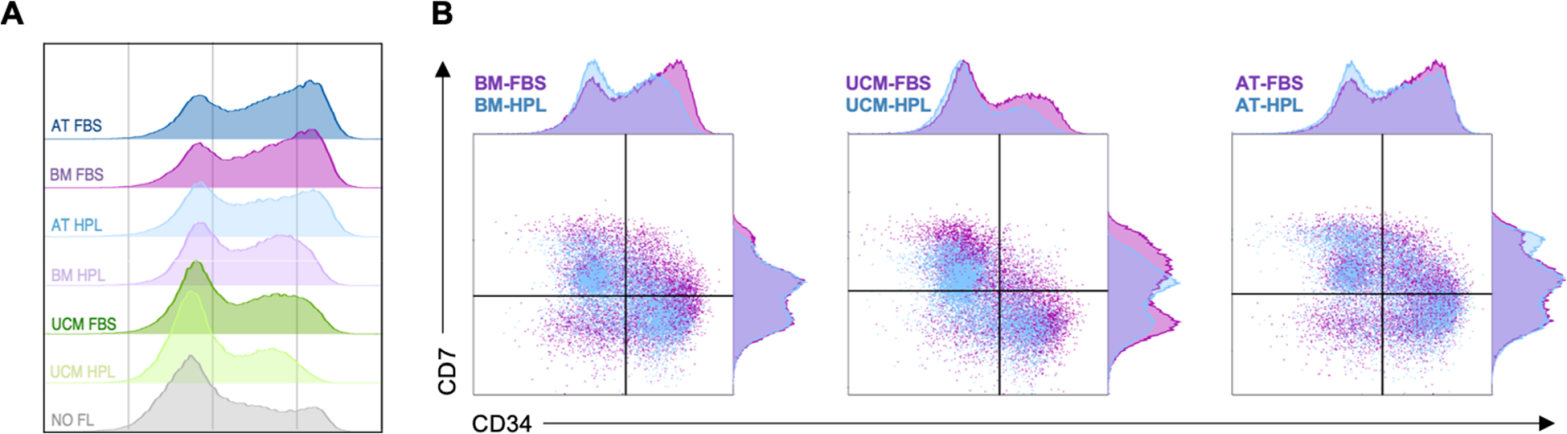
Comparison of CD34 and CD34/CD7 expression by expanded umbilical cord blood (UCB) cells after 7 days of co-culture with mesenchymal stromal cells (MSC) from different sources previously established using different medium supplementation (fetal bovine (FBS) or human platelet lysate (HPL)). **a** Histogram overlay of CD34 expression by expanded UCB cells co-cultured with MSC feeder layers from different tissue sources that were previously established with FBS- or HPL-supplemented medium (representative UCB donor). **b** Dot plot overlay of CD34 and CD7 co-expression by expanded UCB cells co-cultured with MSC feeder layers from the same tissue source that were previously established with FBS- or HPL-supplemented medium (representative UCB donor). AT, adipose tissue; BM, bone marrow; FBS, fetal bovine serum; HPL, human platelet lysate; UCM, umbilical cord matrix

Through immunophenotypic characterization of the expanded and non-expanded UCB-derived HSPC, different dynamics were observed according to the culture medium in which MSC FL had been previously established (Fig. 3). At day 7, a CD34^+^ cell population was maintained in culture, which varied between 30.4 and 69.1% for the co-cultures, with the negative control (NO FL) presenting a population of 43.7 ± 7.0%. Regarding this CD34^+^ cell population, not only differences between the distinct MSC sources were observed, but also between MSC-FBS and MSC-HPL FL within the same source, with the former presenting a higher CD34^+^ cell content: 69.1 ± 2.3% (BM-FBS) vs 56.2 ± 0.8% (BM-HPL) (p < 0.05); 45.4 ± 2.9% (UCM-FBS) vs 30.4 ± 3.2% (UCM-HPL) (p < 0.01); and 68.6 ± 0.9% (AT-FBS) vs 56.1 ± 2.2% (AT-HPL) (p = 0.056) (Fig. 4a). In order to highlight the differences observed in what concerns CD34 expression by the expanded UCB in the different culture conditions, we performed a histogram overlay analysis (Fig. 5a). For all conditions tested, two peaks can be observed: one corresponding to CD34^+^ cells (positive peak) and the other to CD34^-^ cells (negative peak). The positive peak (i.e., CD34^+^ cells) is notably higher and more pronounced for the conditions where BM-FBS and AT-FBS FL were used. For UCB cells expanded in BM-HPL and AT-HPL conditions, the positive peak is not as high or intense as observed for the FBS conditions. Concerning the UCB cells expanded with a FL of UCM MSC, the positive peak is the smallest of all co-cultures, being smaller and less intense for the UCM-HPL condition than the UCM-FBS condition, resembling the behavior observed with HSPC expanded under feeder-free conditions (NO FL). In order to depict the more primitive stem/progenitor content of the UCB HSPC after culture, the immunophenotypic analysis included the assessment of CD34 and CD90 surface markers, as the subpopulation containing more primitive cells is characterized by its simultaneous expression (i.e., CD34^+^CD90^+^ cells). While BM and AT FL were able to maintain a similar percentage of this specific subpopulation of more primitive UCB HSPC, the UCM FL seems to have yielded a smaller percentage. Nonetheless, for all MSC sources studied, the percentage of this subpopulation showed a tendency to be inferior when MSC-HPL were used in the co-culture system: 3.2 ± 1.7% (BM-FBS) vs 2.0 ± 1.0% (BM-HPL); 2.9 ± 1.1% (AT-FBS) vs 2.3 ± 0.6% (AT-HPL); 1.5 ± 0.3% (UCM-FBS) vs 0.9 ± 0.3% (UCM-HPL); and 0.6 ± 0.3% (NO FL). Another subpopulation of interest that was assessed were the proT cells (i.e., progenitor T cells), defined as cells that co-express the surface markers CD34 and CD7 (i.e., CD34^+^CD7^+^ cells). For the proT cell subpopulation, the immunophenotypic analysis showed the same pattern observed in the subpopulation containing more primitive cells. Of notice, the difference between HPL and FBS conditions is also evident for the proT cell subpopulation, as UCB HSPC that were co-cultured with BM-FBS resulted in an increase of more than 50% on the expression of both CD34 and CD7 markers, when compared to BM-HPL: 32.1 ± 2.9% vs 19.3 ± 3.2% (p < 0.01). Co-cultures using the other MSC sources followed the same tendency: 33.4 ± 4.2% (AT-FBS) vs 20.2 ± 1.0% (AT-HPL); 8.9 ± 0.7% (UCM-FBS) vs 4.1 ± 0.9% (UCM-HPL); and 10.1 ± 3.8% (NO FL) (Fig. 4a; Fig. 5b). Expansion levels of the aforementioned subpopulations (CD34^+^, CD34^+^CD90^+^ and CD34^+^CD7^+^), expressed as FI, followed the tendency observed for cell population content (in percentage) (Fig. 4b).

In all cultures, the differentiative potential of the expanded UCB cells was mainly shifted towards the myeloid lineage (Fig. 4c). A high expression of CD33, a myeloid-specific receptor, was observed on non-expanded cells (day 0), but also on expanded cells regardless of the MSC source used in the co-culture system, as well as on the control without FL. Nonetheless, UCB cells expanded over MSC-HPL seem to show a slight decrease of this marker compared to MSC-FBS conditions. CD14^+^ cells (monocytic potential) were also present on day 7 (11.0–16.4%), without any obvious differences between UCB cells expanded over MSC-FBS or MSC-HPL within each MSC source. On the other hand, UCB cells expanded without FL showed a residual population with monocytic potential (1.3 ± 0.2%). Conversely, hematopoietic cells expanded under feeder-free conditions (NO FL) displayed a considerable population (10.7 ± 4.9%) of CD41α^+^ cells (megakaryocytic potential) whereas the CD41α^+^ population varied between 2.2 and 3.5% in the co-cultures. CD15^+^ cells (granulocytic potential) were also present among the expanded UCB cells and its percentage varied according to the MSC source used to establish the FL (6.3-11.5%), with no evident influence of culture medium used; UCB cells expanded under feeder-free conditions (control) comprised a population of 8.0 ± 1.0% CD15^+^ cells.

### UCB expanded cells maintain their clonogenic potential regardless of the MSC tissue source and culture conditions

**Fig. 6:**
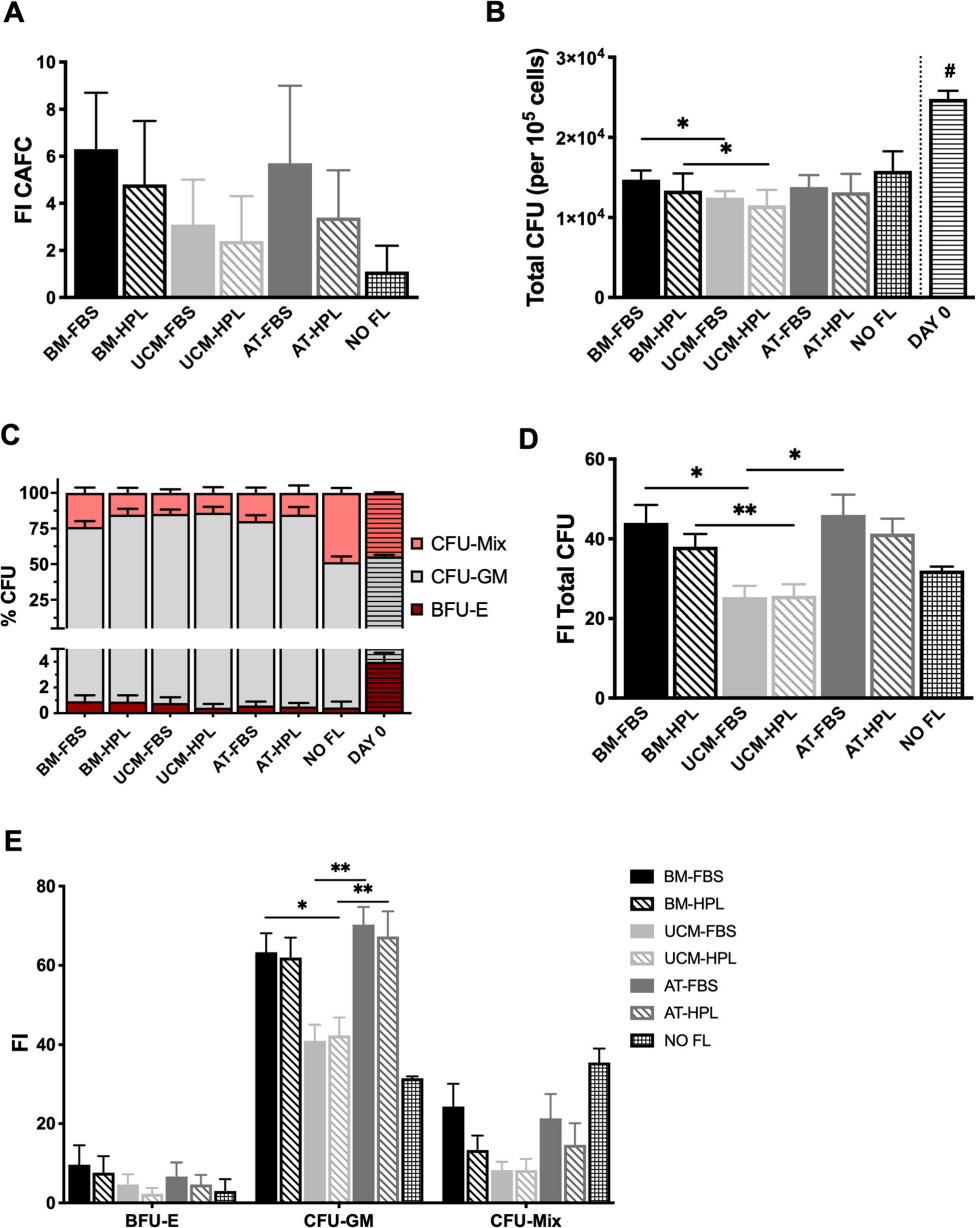
Cobblestone area-forming cells (CAFC) and colony-forming unit (CFU) produced by hematopoietic stem/progenitor cells (HSPC) before and after a 7-day expansion in a co-culture system with mesenchymal stromal cells (MSC) from different sources. **a** Fold increase of CAFC. **b** Total CFU obtained per 10^5^ UCB cells. **c** Percentage of each CFU type (BFU-E, CFU-GM, CFU-Mix) produced. **d** Fold increase of total CFU. **e** Fold increase of each CFU type (BFU-E, CFU-GM, CFU-Mix). AT, adipose tissue; BFU-E, erythroid burst-forming unit; BM, bone marrow; CFU-GM, colony-forming unit granulocyte-monocyte; CFU-Mix, multilineage colony-forming unit; FBS, fetal bovine serum; FI, fold increase; FL, feeder layer; HPL, human platelet lysate; UCM, umbilical cord matrix. *n* = 3 for all conditions, with exception of ‘NO FL’: *n* = 2. Values are presented as mean ± SEM. **p < 0.01; *p < 0.05; #*p* < 0.01 vs BM-FBS, UCM-FBS, UCM-HPL, AT-FBS and *p* < 0.05 vs BM-HPL, AT-HPL

Besides immunophenotyping, we performed two different assays to identify the presence of primitive cells and the clonogenic potential of the progenitor cells: the cobblestone area-forming cells (CAFC) assay and the colony-forming unit (CFU) assay, respectively. By using our co-culture expansion system, we verified that, regardless of the MSC source used to expand the UCB cells, all of them allowed a FI in the number of CAFC of expanded UCB cells (Fig. 6a). There seemed to a be a tendency for a FL of BM or AT MSC to allow for a slight increase in the FI, as the mean ranged from 3.4 to 6.3, while the mean FI of CAFC of the expanded cells ranged between 2.4-3.1 when a FL of UCM MSC was used. However, no statistical significance was found among MSC sources. UCB cells expanded without a FL (NO FL) presented the smallest mean FI of CAFC (1.1 ± 1.1).

Regarding the clonogenic potential, assessed through the CFU assay, as expected, the number of total CFU obtained per 10^5^ HSPC was superior for UCB cells before expansion, on day 0 (2.5 × 10^4^ ± 1.0 × 10^3^), than after expansion (Fig. 6b). After the 7-day expansion in the co-culture system, even though the CFU values obtained with BM-MSC and UCM-MSC slightly differed between them, overall the number of total CFU was similar for UCB cells expanded in any of the MSC sources, ranging from 1.2 × 10^4^ ± 1.9 × 10^3^ (UCM-HPL condition) to 1.5 × 10^4^ ± 1.2 × 10^3^ (BM-FBS condition). The culture medium in which MSC were established did not seem to have impact on the CFU total number produced by the UCB cells. Interestingly, cells expanded without a MSC FL (NO FL) seemed to show a higher number of total CFU (1.6 × 10^4^ ± 2.5 × 10^3^) than the ones expanded with a MSC FL. We also looked in detail to the type of CFU produced (Fig. 6c), namely erythroid burst-forming unit (BFU-E), colony-forming unit granulocyte-monocyte (CFU-GM) or multilineage colony-forming unit (CFU-Mix). For non-expanded cells (UCB cells on day 0), we verified that half of the colonies presented were committed to the myeloid lineage, as 51.5 ± 0.5% of the colonies were CFU-GM, but there was also some erythroid potential, seen not only by the percentage of BFU-E (4.0 ± 0.7%) but also CFU-Mix (44.5 ± 0.5%). For the UCB cells expanded in a co-culture system, regardless of the MSC source used, we observed a shift in the type of colonies produced, as the great majority (> 70%) were CFU-GM colonies. The erythroid potential was reduced, as less than 1% of the colonies were BFU-E, with the remaining colonies being CFU-Mix. Interestingly, for the conditions where UCB cells were expanded with MSC-HPL, the percentage of CFU-GM colonies showed a tendency to be slightly greater when compared with MSC-FBS conditions: 83.8 ± 4,1% (BM-HPL) vs 75.1 ± 4.1% (BM-FBS) and 84.2 ± 5.5% (AT-HPL) vs 79.6 ± 4.1% (AT-FBS). For the UCM MSC source, similar results were found 85.7 ± 4.1% (UCM-HPL) vs 84.5 ± 3.0% (UCM-FBS). Of notice, UCB cells that were expanded with NO FL presented percentages of CFU-GM and CFU-Mix very similar to the non-expanded cells on day 0, with a reduction in the percentage of BFU-E.

In terms of FI of total CFU, which considers the aforementioned results and the expansion potential of our expansion systems, we verified that it follows the pattern observed for FI in TNC (Fig. 2). Specifically, UCB cells expanded over a FL of BM or AT MSC presented the highest FI in total CFU (~40x), while the results obtained with a FL of UCM MSC were significantly lower (~25x). No significant differences were observed regarding the establishment of the MSC sources with FBS- or HPL-supplementation (Fig. 6d). The influence of the MSC source on the expansion potential of UCB cells in what concerns each CFU type (BFU-E, CFU-GM, CFU-Mix) obtained was also explored (Fig. 6e). In co-cultures with MSC, the highest FI was observed for the CFU-GM, followed by the CFU-Mix and lastly the BFU-E. Although with small differences, BM and AT MSC FL resulted in similar FI of each CFU type for the expanded UCB cells, higher than the ones obtained with UCM MSC. Regarding the culture medium used in the MSC expansion, no influence in the FI of each CFU type seems to exist. On the other hand, expansion of HSPC without a MSC FL allowed for a substantial FI of CFU-Mix, which was very similar with the FI of CFU-GM, in opposition to what was observed for the MSC conditions.

## Discussion

Wide application of umbilical cord blood (UCB) hematopoietic stem/progenitor cells (HSPC) to treat malignant and non-malignant diseases in a hematological context, specifically adult patients, is hampered by the low cell quantity in a single unit. While great efforts are being made to overcome this hurdle, expanding UCB HSPC ex vivo with the support of a bone marrow (BM) mesenchymal stromal cell (MSC) feeder layer (FL) is a relevant and commonly used strategy. Indeed, in 2012, the results of a phase I/II clinical trial where 31 patients with hematologic cancers received transplants of two UCB units, one of which containing HSPC expanded ex vivo in a co-culture system with BM MSC, were published [13]. Since transplantation of both UCB units proved to be safe and effective, significantly improving the engraftment compared to unmanipulated UCB only, the study moved to a phase III clinical trial. The study is now completed, but the results were not yet published (ClinicalTrials.gov Identifier: NCT01854567). Despite the encouragement and great advances that these clinical trials present to the field, they also mirror the need for optimization of these co-culture systems. Culture period is one of the topics that should be optimized. By doing a 2-week expansion of the UCB cells in the co-culture system with MSC, a medium change over the first week is demanded, which entails a superior cost of reagents, time, and handling. Our expansion protocol was designed to last one week without any media changes or handling in between, making it a more efficient system. Additionally, our expansion system, originally established with BM MSC, has its cytokine cocktail optimized for the maximum expansion without compromising the maintenance of HSPC [12], reducing overall cost of goods. Regarding the two main outputs evaluated in the translation of an expansion protocol to a clinical trial, i.e., the fold increase (FI) of total nucleated cells (TNC) and CD34^+^ cells, the authors were able to reach a FI of 12.2 in TNC, while the CD34^+^ cell fraction had a FI of 30.1, after 14 days. In our study, by using a BM MSC FL in co-culture with the UCB HSPC, we were able to obtain a FI of 73.8 and 51.5, respectively, after 7 days. This increase can easily be explained not only by our optimized co-culture system, but also due to the starting cell product: in this clinical trial [13] mononucleated UCB cells were used, while we started with a highly purified population of UCB CD34^+^ cells.

In this clinical trial, off-the-shelf BM-derived MSC isolated using fetal bovine serum (FBS) were used to prepare the FL for the co-culture system. However, MSC isolated from the BM present some disadvantages related to the source per se, since BM collection is a very invasive procedure that entails risks for the donors. In addition, BM collection is not done as frequently as peripheral blood stem cell mobilization and collection, thus decreasing the availability of this source. While the majority of expansion studies use BM MSC derived from the iliac crest of the hip bone (from HSPC donors in a hematopoietic cell transplantation (HCT) context), BM samples can also be obtained from routine bone surgeries, namely from knee surgery. However, these medical interventions are normally associated with elderly patients, and it is well known that a decrease in MSC functionality, as well as in the number of passages they are able to withstand, is correlated with an increase in donor age [35]. Thus, although hematopoiesis occurs in the BM and from the physiological point of view the use of BM MSC would be the rational choice, alternative sources are required to ease clinical translation. Fortunately, there are other sources of MSC readily available, namely adipose tissue (AT) and umbilical cord matrix (UCM), which are commonly discarded as medical waste. Either of these MSC sources can be collected to isolate off-the-shelf MSC, in a similar way to what is done with BM-derived MSC. Direct comparison of using MSC from different sources, namely BM, UCM, and AT, have already been explored in different areas, such as treatment of graft-vs-host disease (GVHD) [36] and immune cell suppression capacity [37]. However, to our best knowledge, no study has performed a comprehensive comparison of the hematopoietic support provided by different sources of MSC to an enriched population of UCB HSPC.

In our study, we directly compared how three different sources of MSC—BM, UCM, and AT—influenced the expansion of UCB-derived CD34^+^ cells. This highly enriched population was isolated from cryopreserved mononuclear cells (MNC) of UCB samples. By using cryopreserved samples, we are replicating what is nowadays commonly performed in a clinical setting, since UCB transplants are routinely performed with cryopreserved UCB units. Moreover, since the goal is to create an expansion product that can surpass the cell number issue of a single unit, studies than can achieve so by using cryopreserved samples are closer to reality. Additionally, we went further by exploring a serum-free (SF)/xenogeneic-free (XF) co-culture system, i.e., we compared the expansion capacity and cell profile of expanded UCB cells over a MSC FL previously established using either FBS or fibrinogen-depleted human platelet lysate (HPL).

By using a FL of MSC that were previously expanded in culture medium supplemented with FBS (BM-FBS, UCM-FBS, AT-FBS) in our co-culture systems, we were able to verify that after seven days of expansion a FI TNC of more than 70 or 80 for BM and AT, respectively, was achieved. Although one could speculate that the tendency for a higher expansion capacity obtained with AT would be accompanied by a loss of hematopoietic progenitors, the immunophenotypic analysis showed the maintenance of a high population (> 65%) of hematopoietic progenitors (CD34^+^ cells) for both BM and AT sources. Concerning the fraction of expanded hematopoietic cells that suffered a loss of their CD34 expression, a shift towards the myeloid lineage was primarily observed (i.e., expression of CD14, CD15, CD33 and CD41α), as previously described by our group [10]. This behavior was substantiated by a higher percentage of CFU-GM in the co-culture system, regardless of the MSC FL source used. The absence of a FL during ex vivo expansion originated similar percentages of CFU-Mix and CFU-GM. This disparity regarding different CFU populations between expansion systems with and without a MSC FL has been previously observed by our group [12]. Interestingly, a small population that contains more primitive cells (CD34^+^CD90^+^ cells) [38] was also kept in both BM-FBS and AT-FBS conditions, which is aligned with the tendency for higher CAFC-producing capacity observed for these conditions. The CAFC assay is a variant of the long-term culture-initiating cells (LTC-IC) assay that does not require cell replating. In our study, it was used to characterize the more primitive compartment of our expanded HSPC population, as this in vitro assay has been proposed as a valid surrogate measurement of hematopoietic reconstitution potential [39]. Our expansion protocol also expanded a particular subpopulation of progenitors that simultaneously express CD34 and CD7, classically defined as proT cells, i.e., progenitor cells with ability to homing the thymus and differentiate into T cells [40]. The expansion of this particular subpopulation is very promising, because not only the myeloid progenitors are being expanded in our co-culture system (as in most ex vivo expansion protocols), but also these precursors of the lymphoid lineage, which are candidates for adoptive T cell therapies [41]. Also, the significant presence of proT cells in our expanded population pool will contribute towards improving the recovery of a potential HCT patient, who inevitably is immunocompromised after treatment. An expanded product with such progenitors capable of a faster lymphopoiesis is critical to reduce HCT-related mortality [42, 43]. Both BM-FBS and AT-FBS FL allowed a percentage doubling for this particular UCB cell population after the expansion protocol. Importantly, and as seen in our previous work of 2010 [10], the presence of a MSC FL showed to be essential for the expansion of proT cells, as in the feeder-free system the percentage of this population was inferior to non-expanded cells. Of note, a population of CD34^-^CD7^+^ cells was also present and expanded using any of the MSC FL. While this expanded population can be differentiated into natural killer and dendritic cells [44] that can be used in cellular immunotherapy, a small percentage of this population has been reported to be able to engraft the thymus in vivo [40], resulting in a broader redefinition of proT cells and suggesting that this population in our expansion system can also be of interest. On the other hand, using a UCM-FBS FL resulted in a lower FI TNC (50) that was also accompanied by a decrease in the progenitor populations seen through CD34^+^ expression (< 50%). Understandably, this decrease in the progenitor populations is also reflected on specific subpopulations, such as proT cells. Although present, the percentage of this population was smaller than before expansion. Compared to the adult sources, UCM MSC appear to provide less hematopoietic support in the conditions of our study, shown by the reduced expansion capacity and also progenitor population maintenance. Of notice, UCM MSC FL were subjected to a higher Mitomycin-C concentration in the growth-arrest treatment compared to their adult counterparts (5 vs 0.5 μg/mL) due to the higher in vitro proliferation capacity of these cells [23]. Mitomycin-C has been commonly employed to inhibit MSC proliferation, as an alternative to irradiation, for multiple assays (e.g., immunomodulatory [45–47], FL for embryonic stem cells [48], induced pluripotent stem cells [49], as well as for HSPC [50, 51]), and the concentration used varies in a 0.5–50 μg/mL range. Importantly, it has been previously acknowledged that different cells might present an intrinsic sensitivity to this antibiotic and thus a dose-response curve to this agent has to be established for each cell type of interest [52]. In this context, a previous study demonstrated the higher efficiency of using Mitomycin-C concentrations of 4–8 μg/mL compared to a 0–2 μg/mL range to growth-arrest human UCM MSC in vitro [33]. Although a possible influence of Mitomycin-C cannot be ruled out, the lower performance of UCM MSC FL in supporting UCB HSPC observed in our study follows the trend present in the field. Other groups have demonstrated an inferior hematopoietic support associated to UCM MSC FL, which was shown using Mitomycin-C-treated [50, 51], irradiated [53], and non-inactivated [54] FL.

Despite the fact that MSC have been mostly cultured with FBS-containing media in clinical trials, the risks associated with the usage of this culture medium supplement are well known, namely the risk of xeno-immunization against bovine antigens and the transmission of pathogens. Ethical issues associated with FBS collection, limited availability, and batch-to-batch variability are also concerns to be considered [28]. In the 1980s, HPL successfully started to be used as an alternative to FBS in the culture of several cell lines and is currently used in the manufacturing of MSC for clinical trials [55, 56]. This supplement is rich in potent bioactive mediators, including various chemokines and growth factors [57]. Current HPL formulations are fibrinogen-depleted (not requiring porcine heparin supplementation) and can be gamma irradiated to reduce pathogen content, which highly reduces the risk of transmission of human diseases by known or unknown viruses. In an attempt to make a product more closely available for clinical purposes, we decided to also establish a MSC FL expanded without the use of animal derived components, by using medium supplemented with fibrinogen-depleted HPL. Interestingly, we noticed that the expansion capacity of HSPC was not affected by the change of MSC expansion medium, as the FI TNC was similar for both FBS and HPL conditions within each source. However, a significant decrease in the percentage of CD34^+^ cells between FBS/HPL conditions within each MSC source was observed for all MSC sources, suggesting a shift from hematopoietic progenitors to more differentiated cells when MSC were expanded with HPL. It is worth mentioning that all differences observed resulted from an adaptation process, as MSC had been isolated with medium supplemented with FBS and first expanded with this medium. Interestingly, previous studies have reported that although HPL-supplemented medium improved the proliferation capacity of MSC in expansion, their immunosuppressive properties were inhibited in comparison to MSC expanded with FBS-supplemented medium. Namely, MSC expanded with fibrinogen-rich HPL showed a reduced capacity to prevent T- and NK-cell proliferation [58, 59]. Importantly, another study has shown that fibrinogen depletion from HPL can partially restore MSC immunomodulatory capacities [60]. Here, we could verify that the hematopoietic support provided by MSC can be modulated by the environment in which MSC grow and the source itself is not the only important factor. Considering our results and the absence of comparative studies concerning the hematopoietic supportive capacity of MSC cultured with HPL, further studies are needed.

Few studies exploring the hematopoietic support given by a FL of AT MSC exist. Without using exogenous cytokines, comparable levels of UCB HSPC expansion were attained when using a BM MSC FL or an AT MSC FL. Curiously, CD7^+^ cell percentage was significantly higher using the AT FL, while the CD34^+^ cell percentage was significantly enhanced using a BM FL [20]. Compared to BM MSC FL, a FL of AT MSC, either from mice or human, favored granulocyte differentiation from peripheral blood stem cells (CD34^+^ cells) and the growth of progenitor cells in vitro to a higher extent. It was suggested that this better support could be attributed to chemokine CXCL12, a critical regulator of hematopoiesis, found to be expressed threefold higher in AT MSC than BM MSC [61, 62], even though less than 1% of genes were found to be differentially expressed between AT and BM MSC [63]. By seeding UCB MNC upon an AT MSC FL and making successive removals of non-adherent cells, Andreeva and colleagues [64] were able to verify that AT MSC enabled the selection of functionally active CD34^+^ HSPC at normoxia (20% O_2_) and hypoxia levels (5% O_2_) after 7 days of expansion. Even though they used an interesting strategy to enrich CD34^+^ cells during culture, their expansion levels were quite low (6-10x) compared to our FI of CD34^+^ cells (60x), which can easily be explained by their lack of exogenous cytokines besides using an expansion system that still relies on FBS usage.

UCM MSC, namely Wharton’s jelly MSC, have also been studied regarding hematopoietic support to UCB CD34^+^ cells. In order to simulate the growth of HSPC in vivo, Zhao and collaborators [24] studied the influence of oxygen percentage in the co-culture system without adding any cytokines, finding out that normoxia values enhanced FI of TNC (3x), CD34^+^ cells and CFU. Although hypoxia levels of 1% O_2_ did not allow the expansion of TNC, they were able to maintain a higher percentage of CD34^+^ cells. By changing the expansion medium from H5100 medium to StemSpan medium supplemented with SCF, FLT3-L, and TPO cytokines, this group attained a higher FI in TNC (>300x), as well as in CD34^+^ cells (90x) [65]. Although similar to our co-culture system, we were able to maintain a higher percentage of CD34^+^ cell population, despite the lower FI in this population. Nevertheless, these differences could be explained by their higher period of expansion (10 days). When Klein and colleagues [54] directly compared MSC from amnion, chorion, and Wharton’s jelly to BM MSC, they verified that a FL of the latter source was significantly superior in expanding UCB CD34^+^ cells. Although their approach was different, as they started the expansion with unfractioned MNC cultured in medium supplemented with FBS over 14 days, BM MSC were shown to be a better source over UCM MSC, which is coherent with our results.

Overall, HSPC ex vivo expansion through co-culture with MSC can be influenced by multiple experimental variables. Whether by using different expansion media, oxygen levels or starting HSPC population, as well as usage of exogenous cytokines, the resulting expansion outcome will inevitably vary. While MSC donor variability could also be considered an important experimental parameter, its impact on the robustness of co-culture HSPC expansions can be controlled. Similar to the manufacturing model for Alofisel, an approved expanded AT MSC-based cell therapy, we expect that a cell bank produced from a single donor will be able to provide innumerous cell doses for MSC FL production. Additionally, by introducing AT as an alternative MSC source, donor availability for co-culture expansions will be significantly improved. Both these points will facilitate the definition of MSC donor acceptance criteria, contributing towards process standardization and current good manufacturing practices (GMP) compliance.

Ultimately, by aiming at the production of clinical-grade expanded UCB HSPC, we envision exploring the scalability of the co-culture system using AT MSC to evaluate the feasibility of attaining numbers with clinical significance. Our co-culture system is currently limited to a 2D surface and performed in static conditions. Translating the co-culture setting into a 3D environment (e.g., using a scaffold for MSC anchorage) and developing a bioreactor to introduce dynamic flow could help improve its scalability and overall viability in producing a potential approved cell-based product. At the same time, it would be important to investigate and clarify the differences and/or similarities behind the supportive hematopoietic capacity of each MSC source at a cellular level. If they could be tracked to individual cell features (e.g., MSC-derived soluble cytokines, extracellular vesicles, adhesion molecules, extracellular matrix molecules, or other [66]), we could potentiate their effect by bioengineering it in a novel expansion system.

## Conclusions

The capacity of mesenchymal stromal cells (MSC) derived from different sources (bone marrow (BM), umbilical cord matrix (UCM), and adipose tissue (AT)) to support the expansion/maintenance of umbilical cord blood (UCB) hematopoietic stem/progenitor cells (HSPC) was directly compared in this study. Our results showed that UCB CD34^+^-enriched cells were better expanded, while preserving the stem/progenitor content, over a feeder layer (FL) of MSC derived from AT. Of note, in addition to myeloid committed cells (e.g., CD33^+^, CD14^+^, CD41α^+^ and CD15^+^ cells), a substantial population of progenitor T cells (CD34^+^CD7^+^ cells) was also maintained and expanded. On the other hand, the expansion capacity of UCB cells was significantly decreased when expanded over a UCM-derived MSC FL. We went further by exploring if MSC FL established using serum-free (SF)/xenogeneic-free (XF) conditions, i.e., using fibrinogen-depleted human platelet lysate (HPL) instead of fetal bovine serum (FBS), would impact the already established SF co-culture system. While the expansion capacity was not affected by this alteration, we noticed a shift from hematopoietic progenitors to more differentiated cells. Still, further studies are needed to fully understand the impact of using HPL (instead of the commonly used FBS) in MSC FL establishment in what concerns its ability to support human HSPC in vitro.

Overall, our study provides important insights concerning the possibility of expanding UCB HSPC in a co-culture system with MSC, derived from other more accessible sources than BM and in a SF/XF context, paving the way towards clinical translation. Also, the developed protocols used herein show a high compatibility with current good manufacturing practices (GMP), since few adjustments would be needed, including the use of the CliniMACS platform (for CD34^+^ cell purification) and the incorporation of a clinical-grade XF hematopoietic expansion medium until a suitable fully chemically defined version can be adopted.

## Data Availability

Please contact the corresponding author for data requests.

## Abbreviations

A/A: Antibiotic-antimycotic
AT: Adipose tissue
BFU-E: Erythroid burst-forming unit
BM: Bone marrow
CAFC: Cobblestone area-forming cells
CFU: Colony-forming unit
CFU-GM: Colony-forming unit granulocyte-monocyte
CFU-Mix: Multilineage colony-forming unit
DMEM: Dulbecco’s modified Eagle’s medium
EDTA: Ethylenediamine tetraacetic acid
FBS: Fetal bovine serum
FI: Fold increase
FL: Feeder layer
GMP: Good manufacturing practices
GVHD: Graft-vs-host disease
HCT: Hematopoietic cell transplantation
HPL: Human platelet lysate
HSPC: Hematopoietic stem/progenitor cells
iBB: Institute for Bioengineering and Biosciences
ISCT: International Society for Cell & Gene Therapy
IST: Instituto Superior Técnico
LTC-IC: Long-term culture-initiating cells
MACS: Magnetic-activated cell sorting
MNC: Mononuclear cells
MSC: Mesenchymal stromal cells
PBS: Phosphate-buffered saline
SCERG: Stem Cell Engineering Research Group
SF: Serum-free
SVF: Stromal vascular fraction
TNC: Total nucleated cells
UCB: Umbilical cord blood
UCM: Umbilical cord matrix
USA: United States of America
XF: Xenogeneic-free
